# The Use of Digital Health Services Among Patients and Citizens Living at Home: Scoping Review

**DOI:** 10.2196/44711

**Published:** 2023-03-27

**Authors:** Milla Rosenlund, Ulla-Mari Kinnunen, Kaija Saranto

**Affiliations:** 1 Department of Health and Social Management University of Eastern Finland Kuopio Finland; 2 The Finnish Centre for Evidence-Based Health Care: A Joanna Briggs Institute Centre of Excellence Helsinki Finland

**Keywords:** health technology, telemedicine, digital, health services, patient care, home, review

## Abstract

**Background:**

The development of digital health services reflects not only the technical development of services but also a change in attitude and the way of thinking. It has become a cornerstone for engaging and activating patients and citizens in health management while living at home. Digital health services are also aimed at enhancing the efficiency and quality of services, while simultaneously providing services more cost-effectively. In 2020, the COVID-19 pandemic accelerated worldwide the development and use of digital services in response to requirements for social distancing and other regulations.

**Objective:**

The aim of this review is to identify and summarize how digital health services are being used among patients and citizens while living at home.

**Methods:**

The Joanna Briggs Institute (JBI) methodology for scoping reviews was used as guidance. A search conducted in 3 databases (CINAHL, PubMed, Scopus) resulted in 419 papers. The reporting was conducted by the Preferred Reporting Items for Systematic Reviews and Meta-Analyses extension for scoping review (PRISMA-ScR), and the analysis of the included papers was performed using a framework consisting of 5 clusters describing the use of digital health services. After screening and excluding papers that did not match the inclusion criteria, 88 (21%) papers from 2010 to 2022 were included in the final analysis.

**Results:**

Results indicated that digital health services are used in different situations and among different kinds of populations. In most studies, digital health services were used in the form of video visits or consultations. The telephone was also used regularly for consultations. Other services, such as remote monitoring and transmitting of recorded information and the use the of internet or portals for searching information, were observed as well. Alerts, emergency systems, and reminders were observed to offer possibilities of use, for example, among older people. The digital health services also showed to have potential for use in patient education.

**Conclusions:**

The development of digital services reflects a shift toward the provision of care regardless of time and place. It also reflects a shift toward emphasis on patient-centered care, meaning activating and engaging patients in their own care as they use digital services for various health-related purposes. Despite the development of digital services, many challenges (eg, adequate infrastructure) still prevail worldwide.

## Introduction

The use of digital health services has become increasingly relevant for health care professionals, patients, and citizens as the COVID-19 pandemic has challenged the health sector worldwide [1-3]. However, what we consider as digital health has evolved over time. At the time that Frank [4] first introduced the concept, digital health was considered mainly in terms of internet-based functions, such as finding information on the internet, or as a means of health e-commerce and also as internet-based applications for integrating information from different information systems. In 2001, Eysenbach [5] used the term “eHealth” not only to mean that health services and health-related information are delivered or enhanced using information and communication technology (ICT) but also in a wider sense as a networked way of improving health care with the help of ICT. Eysenbach [5] stated that eHealth is not just about the technical development of services but also about the development of different attitudes and ways of thinking. Eysenbach [5] presented the 10 e’s (eg, efficiency, evidence based, empowerment, encouragement, ethics, and equity) that are inseparable from the concept of eHealth. Since then, further clarification and updating of the term “eHealth” have been called for [6].

Today, the term “digital health” encompasses many other technologies than just internet-based solutions. In addition to digital health, terms such as “digital health services,” “eHealth,” and “telemedicine” are used with slightly different meanings [7]. These solutions not only include internet-based ICT solutions but also other types of technologies, such as artificial intelligence, wearables, and mobile apps. The World Health Organization (WHO) considers digital health services as a secure and cost-effective use of ICT for providing access to health and health-related fields, such as health surveillance, education, knowledge, and research. [8] The European Commission (EC), in contrast, emphasizes the concept of digitalization and considers digital health services as either partly or fully digitalized by using digital elements and solutions to provide health services. According to the EC [9], digitalization is not only a technical but also an organizational and cultural process. In this review, digital health services are considered in their broad concept, covering all kinds of technology solutions used for delivering health care services digitally.

The development of digital services in health care plays an important role in involving individuals in managing their health and maintaining activity in managing their health and overall well-being [10,11]. This can be described as a paradigm shift toward participatory medicine, of which a cornerstone is full patient access to their medical records [12,13]. The paradigm shift from traditional to modern medicine enhances shared decision-making between the patient and the health care professional as well as democratization of care, leading thus to a more equal patient–health care professional relationship [13]. To be able to participate actively in decision-making, patients need health literacy skills that enable them to obtain and understand health information and share their preferences, values, and experiences with health care professionals [14].

In addition to activating patient participation, the development of and the increase in digital health services are aimed at enhancing the efficiency and quality of services and providing services more cost-effectively from the service provider's point of view. Between the customer and the service provider, digital health services, such as patient portals, provide a completely new opportunity for arranging care regardless of time and place [5,15,16]. The value of care is created and defined in terms of meeting the patient’s needs and thus affecting the quality and cost-effectiveness of the care and the performance of the health care provider [17].

The use of digital health services depends on many factors [18,19]. Patients and customers possibly have positive attitudes toward using digital services, especially when having positive perceptions of the usefulness and ease of use of digital health services [20]. Even among elderly people, satisfaction with and the preparedness to use digital health services have been observed [21-26]. According to studies conducted during the pandemic, patients stated that they were willing to continue using digital health services even after the pandemic [27].

Digital health services can include many examples of solutions for patients and citizens. In this review, digital health services refer to all possible technology-based solutions that enable health management while living at home. These solutions include technologies operated via computers, tablets, and mobile phones, as well as wearable and monitoring software for measuring and collecting data on the user’s health [28]. The definition of health is more complex. In 1946, WHO [29] defined health as a “state of complete physical, mental, and social well-being and not merely the absence of disease or infirmity.” Later, WHO [30] expanded the definition to also mean a resource for life and for the continuous process of people to promote their health. Due to the complexity of the definition and its implications, for example, health policy and services, new definitions are required [31,32]. In this review, health is considered in its broader context, as defined by WHO [30].

As the use of various technologies is becoming more common in health care, more patient engagement and activation are required. In this setting, activation of patients refers to not only knowledge and skills but also confidence to manage one’s health. This is considered a prerequisite for a patient to make informed choices concerning their care [11]. This scoping review aims to explore the publications published since 2010 studying the use of different kinds of digital health services among patients and citizens living at home. The review’s focus is solely on technology solutions that can be used in the home environment, thus highlighting the various possibilities of digital health services.

## Methods

### Design

This scoping review was conducted using the methodological framework of the Joanna Briggs Institute (JBI) [33]. A scoping review approach can be chosen for a range of reasons [34-39]. In this paper, the scoping review method was chosen to map the extent of the literature on this specific topic, to objectively summarize the available evidence, and to identify knowledge gaps and thereby contribute to future research. Based on the reasons for conducting a scoping review, no critically appraised or synthesized answer to the research question is offered; rather, the aim is to provide evidence of the particular phenomenon [39].

### Scoping Review Question

The research question for this scoping review is: How are digital health services used among patients and citizens while living at home?

### Inclusion Criteria

The inclusion criteria were identified in relation to the research question with the help of the Population, Concept, and Context (PCC) framework [33]. The population regarding the research question were all patients and citizens who use digital health services, and the concept was digital health services. In this study, we defined digital health services as any solutions that use different information technologies. In this review, a wide range of study designs, such as randomized controlled trials, cohort studies, cross-sectional studies, and reviews, were considered. Protocols that provide a plan for a review or study were excluded from the review. The context in this review was the home environment; thus, studies in which digital health services were used elsewhere, such as in hospitals or long-term care facilities, were excluded. Studies were also excluded if the use environment was not apparent. In the search, papers published in open access and peer-reviewed scientific journals between January 1, 2010, and March 8, 2022, were retrieved. The search included journal papers published in English, German, or Swedish.

### Search Strategy

The online databases Scopus, PubMed, and the CINAHL were used to retrieve journal papers concerning the use of digital health services among patients and citizens while living at home. The search was conducted on March 9, 2022. The database searches resulted in 152 papers in CINAHL, 28 papers in PubMed, and 239 papers in Scopus.

Keywords related to digital health and the use of digital health services were used to carry out the search. The keywords were *patient*, *customer*, *effectiveness*, *impact*, *effect*, util*, *ehealth*, *digital service*, *electronic health*, *digihealth*, *telehealth*, *telemedicine*, *m-health*, *digital health*, *healthcare*, *health care*, *hospital*, *health*, and *care*. They were used with various combinations using the Boolean operators AND and OR. An information specialist of the University of Eastern Finland assisted in refining the search strategy. The search strategies are presented in Table 1.

**Table 1:** Search strategies.

Database	Search terms
Scopus	( TITLE-ABS-KEY ( patient* OR customer* OR citizen* ) AND TITLE ( use OR usage OR util* ) AND TITLE ( ehealth OR “digital service*” OR “electronic service*” OR “electronic health*” OR digihealth OR telehealth OR telemedicine OR m-health OR “digital health” ) ) AND PUBYEAR > 2009 AND ( LIMIT-TO ( OA , “all” ) ) AND ( LIMIT-TO ( DOCTYPE , “ar” ) OR LIMIT-TO ( DOCTYPE , “re” ) ) AND ( LIMIT-TO ( LANGUAGE , “English” ) OR LIMIT-TO ( LANGUAGE , “German” ) OR LIMIT-TO ( LANGUAGE , “Finnish” ) OR LIMIT-TO ( LANGUAGE , “Swedish” ) OR EXCLUDE ( LANGUAGE , “Portuguese” ) OR EXCLUDE ( LANGUAGE , “Spanish” ) )
PubMed	((((patient*[Title/Abstract] OR customer*[Title/Abstract] OR citizen*[Title/Abstract])) AND (use[Title] util*[Title] OR usage*[Title])) AND (ehealth[Title] OR “digital service”[Title] OR “electronic service”[Title] OR “electronic health”[Title] OR digihealth[Title] OR telehealth[Title] OR telemedicine[Title] OR m-health[Title] OR “digital health”[Title])) AND (health[Title/Abstract] OR well-being [Title/Abstract] OR wellbeing[Title/Abstract])
CINAHL	AB ( patient* OR customer* OR citizen* ) AND TI ( use OR usage OR util* ) AND TI ( ehealth OR “digital service*” OR “electronic service*” OR “electronic health*” OR digihealth OR telehealth OR telemedicine OR m-health OR “digital health” ) AND AB ( wellbeing OR well-being OR health )

### Study Selection and Inclusion

**Figure 1:**
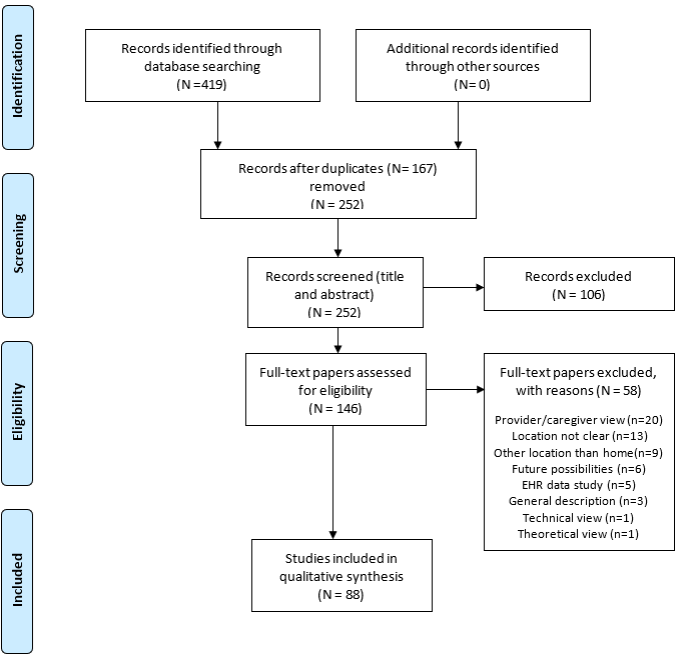
Flowchart of the selection procedure [40]. EHR: electronic health record.

The selection procedure and data extraction were performed by the first author of the paper. The studies were then reviewed and selected in 3 stages. Studies that did not meet the inclusion criteria were excluded at each stage accordingly. Initially, the search in the 3 databases identified 419 papers. The database search results were then uploaded to the ProQuest RefWorks citation manager. After excluding 167 (39.9%) duplicates in the first stage, 252 (60.1%) papers were eligible for further screening. In the second stage, the titles and abstracts of the papers were screened and 106 (42.1%) papers were rejected because they did not meet the inclusion criteria; 146 (57.9%) papers were eligible for full-text review. A full-text review was conducted in the third stage, and finally, 88 (60.2%) papers were selected for this review. The procedure of this scoping review is presented in Figure 1, which is based on the Preferred Reporting Items for Systematic Reviews and Meta-Analyses extension for scoping review (PRISMA-ScR) flow diagram [40].

The main reason for rejection (n=22, 15.1%, papers) based on full-text review was that the papers were from the provider’s or caregiver’s point of view. Other reasons for rejection were that the studies (n=13, 8.9%, papers) did not clearly state whether the use of digital health care services took place at home or elsewhere or that the studies were conducted in a hospital environment, a long-term care facility, or a location other than home (n=8, 5.5%, papers). Further reasons for rejection were that the papers discussed future possibilities (n=6, 4.1%, papers) or dealt with using electronic health record (EHR) data for study purposes or EHR standards (n=5, 3.4%, papers). Additional reasons for exclusion included describing the general use of digital health services (n=3, 2.1%, papers) or describing the use of digital health services from a technical (n=1, 0.7%, paper) or theoretical (n=1, 0.7%, paper) point of view. Characteristics of the papers and extracted data are presented in the Results section and finally concluded in the Discussion section.

### Data Analysis

The final data for this review is presented in alphabetical order in Multimedia Appendix 1. For each included paper, the following information was recorded: author, year of publication, country of origin, objective, study design, population, device and use, and main results. “Device and use” was chosen as the primary theme based on the objective and main research question of this scoping review. The analysis was performed deductively using the framework developed by Harst et al [41]. The framework classifies interventions into 5 clusters: telemonitoring, teleconsultation, telediagnosis, teleambulance/tele-emergency, and digital self-management [41].

## Results

### Characteristics of the Included Papers

Altogether, 88 papers were included in the review, all written in English. Geographically, over half (50/88, 56.8%) of the papers (their first authors) were from the United States [42-91]. Further, 8 (9.1%) papers were from Australia [92-99], 4 (4.5%) each from the Netherlands [100-103] and Germany [104-107], and 3 (3.4%) from Canada [108-110]. China [111,112], India [113,114], Norway [115,116], and Thailand [117,118] were each represented in 2 (2.3%) papers. Other countries represented were Bangladesh [119], the Czech Republic [120], Denmark [121], the United Kingdom [122], Greece and Finland (a joint paper) [123], Italy [124], Jamaica [125], Libya [126], Saudi Arabia [127], South Africa [128], and Turkey [129].

Of all the included papers, 15 (17.0%) were published in JMIR publications, 6 (6.8%) in BioMed Central (BMC) journals, 4 (4.5%) in the *Journal of Telemedicine and Telecare*, 3 (3.4%) in the *Journal of the American Informatics Association*, 3 (3.4%) in *Telemedicine and e-Health*, and 3 (3.4%) in British Medical Journal (BMJ) publications. In addition, 2 (2.3%) papers were published in *Rheumatology Advances in Practice* and 2 (2.3%) in each of the Journal of the American Medical Association (JAMA) publications (*JAMA Network Open* and *JAMA Surgery*) as well as in the *Journal of Substance Abuse Treatment*. The rest of the papers (n=50, 56.8%) were each published in a different journal. In addition, 74 (84.1%) of the 88 papers were published between 2017 and 2022, and 38 (43.2%) of the 88 papers were published in 2021 and 2022 alone, mainly due to the COVID-19 pandemic.

### Characteristics of the Population

Population characteristics of the included papers were classified according to the patients’ age group (pediatric patients, adults, older people). Of the included studies, 35 (39.8%) papers had solely adults (>18 years of age) as participants, 25 (28.4%) papers had both adults and older people as participants, and 8 (9.1%) papers had only older people. However, the definition of older people varied across studies. In 5 (5.7%) papers, participants were pediatric patients. All age groups were represented in 6 (6.8%) papers. The age of the participants was not clearly defined or clearly distinguished in 9 (10.2%) papers.

Most of the participants in the studies had a medical condition that required consultation, surveillance, or monitoring. The most common medical condition was a chronic condition, such as cardiovascular disease, cancer, diabetes, and arthritis. In addition, behavioral health issues and substance use disorders were among the conditions observed in studies. In some studies, health issues related to general medical conditions or no specific health condition was given.

### Use of Digital Health Services

The results of the search showed that the use of digital health services can be extensive and can be used for many different purposes and in different population groups. The results were analyzed according to the methodology given by Harst et al [41], which classifies interventions into 5 clusters, as discussed earlier. In this Results section, the purpose of using digital services is roughly categorized according to the clusters presented in the framework. It should be noted that some of the included studies may overlap across clusters.

Most studies in this review can be included in the teleconsultation and telediagnosis cluster given by Harst et al [41] as the use of digital health services occurred mainly as video (or virtual) visits [42-71,92-96,108,111,113,125,126,128,129] and in some cases led to a diagnosis (eg, Atilgan et al [129]). Examples of the study cases concerning video visits are presented in Table 2.

The examples of video visit usage show that video visits are used in different kinds of populations with varying conditions. A video connection could also be used in combination with patient portals and access to EHRs [42] and different devices and apps [125]. Video consultations offer possibilities for first and follow-up visits at the clinic [46,49,53], for peri- and postoperative sessions [125] and for medication and psychotherapy sessions [59].

In addition to the concept of video visits and videoconferencing, expressions such as video consultation and video encounter were used when patients had consultations with health care professionals via a video connection. In addition, telehealth visits [72,119], platforms [73], eHealth [117], telephone (or voice) calls [45,52,53,63,65,68,73-78,92-98,100, 101,118,127,128], text messages [90], and mobile apps [118] were mentioned as means for consultation. Looi et al [93,94] found that during the second and third quarters of 2021, the telephone was mostly used for telehealth visits in private psychiatry practice in Australia. Whether it is in the form of video visits, telephone calls, or other means, the use of virtual communication has sharply increased due to the COVID-19 pandemic. Many countries still face challenges in implementing widespread use of digital health services according to national and international guidelines [2].

The important advantage of digital health services is the potential to activate citizens and patients to participate, engage more in maintaining their own health, and support shared decision-making between health care professionals and patients [9,41]. Thus, the use of digital health services may play a central role for patients and citizens in self-management of their health. In the Harst et al [41] framework, this refers to a *self-management cluster*. Digital services for self-management among the studies included the use of portals or eHealth platforms [42,44,55,62,98,100,101], online programs, and mobile [75-81] and social media platforms [128]. For example, the patient-reported outcome (PRO) assessment that was conducted via an iPhone was used to support the self-management and clinical decision-making for patients with cancer, which seemed to be highly acceptable among patients [101]. Social media platforms were used, for example, for multifaceted eHealth, including websites, digital monthly newsletters, and social media platforms, among patients diagnosed with nonspecific low back pain, which, however, did not show any effectiveness in improving the patients' back pain beliefs or in decreasing disability and absenteeism [102]. Through different means (internet, portals, etc), citizens and patients can use and explore their personal health records in support of self-management, for example, during and after hospital discharge from cardiac care [82], for obtaining personalized recommendations on actions concerning one’s health [121], or for enabling communication between patients and different health care settings in a prototype study [104]. Personal health records were also seen useful in keeping track of different kinds of health-related needs, such as medications [83].

Digital health services respond widely to the need to acquire information about health-related issues. The internet was used as an important source of health information in several studies [84,92,105,106,109,112,115,116,120,123]. Athanasopoulou et al [123] studied the use of the internet for health-related purposes among Finnish and Greek patients with schizophrenia spectrum disorders and found that the use of the internet for health-related purposes was similar among patient groups. However, Finnish patients considered the internet the second-most important source, while Greek patients considered the internet the least important source of health information [123]. The internet was also used, for example, for online education modules [92], social media and video services [116], and promotion of clinical decision-making [112]. In addition to self-management at home, digital services can be used for remote monitoring. In line with Harst et al [41], this review assigned telemonitoring as a cluster of its own. Remote monitoring has played a central role in opening up possibilities to provide digital health services while patients live at home [48,85-87,99,103,107,114,120,122,124,129]. In these studies, monitoring was mainly used for recording vital parameters and transmitting data from the home to the health care professionals at clinics. Examples of study cases concerning remote monitoring are presented in Table 3.

The examples indicate that remote monitoring can be used effectively at home for different purposes. Not only monitoring vital signs [85,87,99,129] or other values for disease management [114] but also performing, for example, spirometry at home [48,107] show possibilities for using digital services from a distance.

The included studies mentioned to a lesser extent the use of digital services as alarms, alerts (eg, fall alerts) or reminders [83,89,90,97]. In its simplest form, patients can use a reminder to remember their health-related appointments and tasks as well as to take medications [83,97,103,129]. Reminders can also be a functionality of a remote monitoring device [103] as well as notifications of a device [79]. Medication reminders, in contrast, offer other kinds of possibilities, such as improving medication adherence [88]. Personal emergency response systems are used by patients as fall alert systems, at least in the United States, and they include the use of a help push button worn as a necklace or a bracelet, an in-home communication system, and an emergency response center [88]. In their study, Agboola et al [88] found that the use of fall alert systems combined with personal medical records can enable improvement in health outcomes in older patients with chronic medical conditions. These digital services that include alarms, alerts, and reminders can be linked to the teleambulance/tele-emergency cluster, as they rapidly can react to, for example, the patient’s health status, if needed [41].

**Table 2:** Video visit examples by population and use.

Author	Population	Use of video or virtual consultation
Abel et al [42]	Veterans with mental health disorders	In the study, veterans used a patient portal and EHRs^a^ only, a clinical video connection only, or a combination of the 2. One group did not use digital services at all. Digital services were used in the form of video for consultations or visits. The engagement in the use of technology in the study remained low.
Akhtar et al [44]	Emergency department (ED) patients with a sore throat	Video visits were carried out by using a video connection and a flashlight for examining the sore throat. In the study, video visits were judged easier to use for providers than patients.
Chao et al [46]	Surgical outpatients	Patients used video regularly for first visits in surgical specialties in response to the COVID-19 pandemic. The barriers to video visit usage were lack of necessities, such as private space, a stable connection, and a device on which to contact clinicians, and lack of an understanding of how to use a video platform.
Dayal et al [49]	Children with neurologic conditions	Video consultations were used by pediatric patients who received outpatient care from pediatric neurologists. Video consultations were associated with lower hospital use compared to in-person consultations.
Kong et al [53]	Patients visiting a rheumatology clinic	Patients of a rheumatology clinic used a video connection for clinic visits. The barriers to usage were older age, limited access to technology, and a short distance to the clinic.
Lin et al [59]	Patients with substance use disorders	Patients with substance use disorders used a video connection for clinic visits for psychotherapy and medication treatments. The use of a video connection was mostly associated with high patient satisfaction.
McGrowder et al [125]	Patients with breast cancer	For patients with breast cancer, videoconferencing offers an opportunity to be used in the area of teleoncology (eg, Zoom, WhatsApp); for peri- and postoperative sessions, rehabilitation, mental health issues; and for instructing physical exercises.
Powers et al [67]	Patients with dementia and their caregivers	Patients with dementia and their caregivers used a video connection in contact with a dementia or geriatric primary care clinic. Acceptance of using a video connection was high among users and saved thousands of travel miles.

^a^EHR: electronic health record.

**Table 3:** Remote monitoring examples by population and use.

Author	Population	Use of remote monitoring
Atilgan et al [129]	Patients after cardiac surgery	Patients used remote devices to record vital parameters, such as blood pressure, pulse rate, saturation, body temperature, blood glucose, and electrocardiography. The data were stored in web-based and mobile apps and used in follow-up for postoperative outcomes. A total of 144 (6.1%) potentially life-threatening complications were found early in this study using remote monitoring.
Compton et al [48]	Adult patients with cystic fibrosis	Patients performed spirometry at home, and the results were monitored by the clinic. The users got instructions, reminders, and coaching for use during the study. Monitoring spirometry data with home devices remotely was seen as reliable and sustainable. The process was also seen as replicable to other clinics.
Kesavadev et al [114]	Patients with type 2 diabetes	Patients self-monitored glucose and hemoglobin A1c (HbA1c) values and other biochemical measurements. The data were stored and then reported via the telephone, email, or websites before the following consultation. The remote self-monitoring was seen as safe and cost-effective in the treatment of type 2 diabetes.
Kuipers et al [103]	Patients with respiratory diseases	Patients used an electronic inhalation-monitoring device to remind themselves of medications and register inhalations. The device was connected to a mobile app, where the data were stored. The data were additionally linked to an online portal, which was used by health care professionals. The electronic inhalation-monitoring device was found to be acceptable and easy to use, but many hesitated to continue its use. More user-tailored features were desired.
Radhakrishnan et al [85]	Patients with heart failure	Patients were monitored remotely after hospital discharge, and different sets of data was collected. Remote monitoring itself did not seem to affect the likelihood of rehospitalization neither for all-cause hospitalizations nor for cardiac-related hospitalizations.
Sengpiel et al [107]	Outpatients after lung transplantation	Patients used home spirometry for telemonitoring in 1 group storing the data via a Bluetooth-equipped mobile phone. In the other group, home spirometry was used alone without a Bluetooth connection. The use of a Bluetooth connection to store data enabled generating alarm messages. Adherence to home spirometry was 97.2% in the group using Bluetooth and 95.3% in the group using home spirometry alone. Patients using Bluetooth reported less anxiety.
Wade et al [99]	Older adults at risk of being admitted into residential care and their caregivers	Older adults used remote monitoring to measure vital signs, such as blood pressure, heart rate, oxygen saturation level, and body weight, which were sent to their general practitioner. Older people and their caregivers perceived remote monitoring as useful and easy to use.
Yi et al [87]	Medically underserved Black and Hispanic participants	Participants monitored blood pressure with a home blood pressure monitor. The data were transmitted via a modem to a secure database. Remote monitoring was not shown to improve control over usual care in this study. Results indicated that minorities may face barriers, such as restrictions in access to digital services and health resources in general.

Several literature reviews have identified a variety of digital health services and provided insights into using different technologies in health management for patients living at home [59,61,91,92,96,97,110,117,125,128]. These digital health services can belong to different clusters of the framework, for example, specifically teleconsultation and self-management. The reviews have found the use of the telephone, text messages via the telephone, video calls, apps such as WhatsApp, personal health records, and social media and digital and online platforms to be of importance. The use of online education and video consultation and teleconferencing via mobile phone seemed to be useful, for example, for patients undergoing bariatric surgery [92]. The literature review by Kuwabara et al [90] showed that digital technology can be used to improve patient education and skills needed for using digital health services. There are, however, barriers to the usage of digital health services, and the study by Chitungo et al [128] highlighted the infrastructure-related challenges when using digital services.

## Discussion

### Principal Findings

The aim of this review was to identify and summarize evidence on how digital health services are being used among citizens and patients while living at home. The focus of the review is on the patient perspective as the development and use of digital health services are considered a central tool for activating citizens and patients to manage and maintain their own health [10,11]. When Frank [4], some 2 decades ago, first introduced the concept of digital health, the idea was primarily based on the internet and the functions it enabled. The idea of digital health, or eHealth, as Eysenbach [5] named the phenomenon, was then broadened to cover functions that are delivered or enhanced by using ICT in a networked way. Eysenbach [5] pointed as well to the need for a change in mindset and attitude. Since the beginning of the 21st century, the pace of the development and use of technology has been remarkable [4,5]. This has been accompanied by a shift from traditional health care to more patient-centered medicine, which is also seen in the results of this review, as patients and citizens are actively using digital health services and producing information about their health for clinical decision-making concerning their care or voluntarily with the help of remote monitoring devices and transmitting information via portals, apps, or other services, while at the same time continuing their lives at home [10-13,15].

Based on the analysis using the framework presented by Harst et al [41], which consists of 5 clusters, this study found that digital health services are used widely for different health-related purposes. The clusters in the framework include teleconsultation, telediagnosis, telemonitoring, digital self-management, and teleambulance/tele-emergency. Most of the digital health service usage discovered in this review falls into the teleconsultation cluster as the studies involve the use of video or other virtual means of consultation or visits, which partly seems to be because of the COVID-19 pandemic [42-71,92-96,108,111, 113,125,126,128,129]. There are, however, also studies that concurrently fit into the telediagnosis cluster, offering a possibility to access health care using a video connection and thus enabling an early diagnosis [41,57,129]. The results indicate that the use of video consultations will also continue in the future as they are seen as a compatible and cost-effective way of providing consultation also in medical specialties and with different kinds of tools, such as WhatsApp or Zoom [1,2,46,49,53,67,125]. The use of or the possibility for video consultation is especially important for rural and other areas with long distances, though infrastructural or cultural issues may currently prevent or delay the use of digital services in some locations [67,84,108,119]. According to the results, telephone calls were still used frequently for contacting patients [45,52,53,63,65,68,73,74,92-97,127,128]. In some studies, most consultations were done over the phone [74,93,94,128]. For instance, in private Australian psychiatric clinics, short consultations (less than 30 minutes) were conducted mostly over the phone [93,94] at the beginning of 2020. In addition, in sub-Saharan Africa, the use of the telephone played a crucial role in consultations at the beginning of the pandemic despite the many challenges faced in the area [128].

Digital self-management, especially the use of the internet, was, according to the results, found to be important for searching for health information and accessing portals, platforms, websites, web videos, online modules, and web-based programs [84,92,105,106,109,112,115,116,120,123]. The increasing use of the internet and other information sources to search for health-related information highlights the need to promote the development of the skills needed to acquire and understand relevant information [48]. The use of the internet for information searching, video watching, and education module watching requires skills in, for example, eHealth literacy and overall usage skills needed with technology. According to the results, apps were found to be actively used for different kinds of functions, such as transmitting and communicating [55,96,129] or searching for information [112,115], controlling medication [109], obtaining education [91], accessing medical records [55], or managing disease [66,80,91,118]. Apps also enable the collection of PROs among patients with prostate cancer, and in this study, nearly all patients reported that using a smartphone app is easier than or equivalent to paper and pen [79]. Of the various technological solutions used, mobile phones were used in a variety of ways for social media, apps [90,106], text messaging [90], or virtual visits [69].

Remote monitoring of health was used in different kinds of situations. Patients used remote devices, for example, to record 1 or several vital signs [86,87,99,114,129], to perform home spirometry [48,107], to register inhalations [103], to monitor blood glucose data and perform insulin therapy, and to also transmit data to the clinic [124]. Instructions on the use of monitoring devices was given to patients before starting the monitoring at home [103,114,122,124]. The use of remote monitoring devices in collecting and transmitting data has a favorable effect according to the studies on clinical decision-making concerning care, while at the same time, remote monitoring made it possible for patients to live at home [48,99,107,124,129]. Remote monitoring at home seemed to have a positive effect on care as life-threatening situations could be observed early [129], better results for treatment were attained [125,127], and self-management (especially of chronic conditions) was made easier [124]. Remote monitoring was also found to be a reliable, sustainable, and cost-effective part of the care of the patient [48,114]. In nearly all studies, the health care provider was actively involved in the care process of managing and monitoring patient health or health information, such as vital signs [76,99,120,127]. In some cases, however, digital services for remote monitoring did not lead to better results (eg, in care adherence) [85,87,122]. Decision-making was also mentioned separately in a few papers that considered the effect of information obtained via the internet on joint decision-making with health care professionals [105,116].

Digital health services were, according to the studies, used widely in different kinds of population groups ranging from children (eg, [49,51]) to older people (eg, [88,96,112]). Geographically, the studies in this review were concentrated to a great extent in the United States, but European countries, Australia, and China were also represented. Some studies addressed the challenge of less industrialized countries where the infrastructure for the digital health services may not yet be adequate for the vast use of digital health services [126,128]. However, digital health services provide a way to engage patients more actively to participate in their own care by providing new ways for usage (eg, internet, health records, apps, and other technical solutions), which contribute to acquiring health information and building up knowledge on health [12].

Overall, the results of this scoping review indicate that using digital health services offer many options for self-care while living at home. Generic services, such as information searching, can be used more autonomously and for self-management, whereas tailored services can be used more for the consultation and management of specific diseases or conditions. As Harst et al [41] mention, digital services may well fit into more than 1 cluster in their framework. The COVID-19 pandemic has clearly provided an impetus for offering alternative ways to citizens to use services enhanced by new technologies in many sectors, not least in health care [1-3]. These services are mainly independent of time and place and thus promote equity in society by providing services from a distance (eg, in rural areas). However, a certain level of digital infrastructure is needed for the implementation of digital services, which is still lacking in many countries, as reflected also in the studies in this review [126,128]. Sociodemographic factors are a barrier to accessing digital services as well [63,65,87,101]. The digital divide and development disparity of digital health services worldwide is perhaps also mirrored in the geographical distribution of the studies in this scoping review.

### Strengths and Limitations

This scoping review was conducted to identify and summarize how patients and citizens use digital health services while living at home. Specifically, population characteristics, digital services used, and outcomes were identified. The objective of this review was not to evaluate the quality of the evidence but to provide evidence of the literature in 3 databases (Scopus, PubMed, and CINAHL) concerning the use of digital health services in managing patients’ and citizens’ health while living at home. Results from other sources (gray literature), such as books, book chapters, and websites, were not included. Solely open access scientific journal papers were included in this review.

The review focused on digital services used at home by citizens and patients and therefore did not consider the services that patients use in hospitals or home-like environments, such as elderly care homes. The health care provider viewpoint was not the topic of this review, although the provider is actively involved in the care process. As only open access papers were considered, relevant papers and the range of gray literature could have been missed. The search was conducted using specific keywords, search terms, and other inclusion criteria, so relevant documents on this broad topic may have been missed. Health can be considered in a broad or specific sense, but in this review, the concept of health was used as defined by WHO. In this sense, a limitation of this study is that it covered only health care services, as health can be seen (as defined by WHO) as a sum of the physical, mental, and social aspects of one’s well-being. In some papers, issues such as drug and other substance use disorders or alcohol abuse were discussed [72,73]. In the northern European context, these belong primarily within the purview of social services. So maybe using social services as a keyword would have provided more relevant results. For instance, in Finland, substance abuse services belong to general social services under the Social Welfare Act [130]. The analysis in this review is roughly based on the framework of Harst et al [41]. The framework and its clusters do not necessarily provide a fully adequate measure for analysis of a vast range of digital services, which is also noted by Harst et al [41].

The strength of the review lies in its ability to describe how vastly digital health services can be used in different kinds of populations when living at home. The review illustrates various potential user groups and different forms of digital services and, thereby, possibilities for the future development of digital health services. The review points out the importance of information for clinical decision-making concerning treatments and also the need for patients and citizens to acquire skills to search, use, and understand health-related information. The results also indicate that digital health services may not be suitable for all population groups. In many studies, facilitators and barriers affecting the use of digital services have been described, but this was not the main topic of this review. The review also notes that a discussion of the development of more equal distribution of digital services worldwide may have value; however, this was beyond the remit of this review.

### Conclusion

The results of the review note the various possibilities of using digital health services while living at home. The use and further development of digital services still face challenges in many levels. However, patients engaging in their own care while living at home indicate a shift from more traditional health care to a modern era in which care can be provided and managed irrespective of time and place. The use of digital services also indicates a shift to more patient-centered care and engaging the patient as part of the decision-making process concerning their health.

## Appendix

Multimedia Appendix 1 Data of the included studies.

Multimedia Appendix 2 PRISMA-ScR checklist. PRISMA-ScR: Preferred Reporting Items for Systematic Reviews and Meta-Analyses extension for Scoping Reviews.

## Abbreviations

EC: European Commission
EHR: electronic health record
ICT: information and communication technology
JBI: Joanna Briggs Institute
PRO: patient-reported outcome
WHO: World Health Organization
