# Supplementary material for: The Use of Digital Health Services Among Patients and Citizens Living at Home: Scoping Review
Source: J Med Internet Res. 2023 Mar 27;25:e44711. doi: 10.2196/44711 (PMC10131924; doi:10.2196/44711)
Supplement: Multimedia Appendix 1 [file jmir_v25i1e44711_app1.docx]

Data of the included studies

| **Author** | **Objective** | **Study design** | **Population** | **Device and use** | **Main results** |
| --- | --- | --- | --- | --- | --- |
| Abel et al, 2018 USA | To describe adoption and use of My HealtheVet and Clinical Video Telehealth among users with mental health diagnoses. | Retrospective, cross-sectional study | Veterans | Patient portal, video visits | Sociodemographic disparities in access to My HealtheVet and Clinical Video Telehealth and in dual use of the technologies and variation based on types of mental health diagnosis were detected. |
| Adepoju et al, 2022 USA | To examine associations between telemedicine use and race/ethnicity and the influence of other individual- and geographical-level factors. | Cohort study | Patients of federal health centers; general medicine | Video visits | Racial and ethnic disparities in telemedicine use persisted. However, telemedicine improved utilization for African Americans and Hispanics living farther away from the clinic. |
| Agboola et al, 2017 USA | To characterize the healthcare utilization of Personal Emergency Response Systems (PERS) patients. | Retrospective, longitudinal study | Older adults with mainly chronic medical conditions | Personal Emergency Response System, which is used primarily as a fall alert system. | Chronic medical conditions account for most of the healthcare utilization in older patients using PERS. |
| Akhtar et al, 2018 USA | To compare the telemedicine-facilitated physical examination with an in-person examination in emergency department (ED) patients with sore throat. | Prospective, observational, blinded diagnostic concordance study | Emergency department (ED) patients with sore throat | Video visit consultation for sore throat | Telemedicine exhibited poor agreement with the in-person physical examination on the primary outcome of tonsil size, but moderate on coloration of the palate and cervical lymphadenopathy. |
| Almandoz et al, 2021 USA | To examine the association of healthcare utilization (telehealth vs. in-person) and sociodemographic factors among patients with obesity during COVID-19 pandemic. | Retrospective study | Patients with obesity | Telehealth (video, telephone, online portal) use | Telehealth use was detected as widely emerging, but utilization varied by age and ethnicity. |
| Alshareef et al, 2021 Saudi Arabia | To assess the suitability of telemedicine in rhinology cases. Satisfaction of patients was also assessed. | Retrospective study | Patients in the rhinology clinic | Phone call for screening of rhinologic symptoms | Telemedicine seemed to be efficient in management and screening of rhinology cases during the COVID-19 pandemic. |
| Athanasopoulou et al, 2017 Finland/Greece | To examine computer/Internet use for general and health-related purposes, eHealth literacy, and attitudes toward computer/Internet among adults with schizophrenia spectrum disorders in Finland and Greece. | Questionnaire survey | Adults with schizophrenia spectrum disorders in Finland and Greece | Internet for health-related purposes | In both countries more than half of Internet users used the Internet for health-related purposes (FI = 61, 55%, vs. GR = 20, 61%). The eHealth literacy of Internet users was found to be significantly higher in the Finnish group. The Finnish group of Internet users scored higher in eHealth literacy, while the Greek group of never Internet users had higher Interest in computer/Internet. |
| Atilgan et al, 2021 Turkey. | To examine cardiac surgery patients who underwent monitoring of postoperative vital parameters using medical monitoring devices that transferred data to a mobile application and a web-based software. | Remote patient monitoring system recording vital parameters after undergoing cardiac surgery | Patients after undergoing cardiac surgery | Remote monitoring recording of vital parameters, contact via voice messages and video with medical staff, and medication reminders | A total of 144 (6.1%) potentially life-threatening complications were found to have been diagnosed early using the Telemedicine System. |
| Bini et al, 2021 USA | To analyze telehealth utilization before and for 1 year during the COVID-19 pandemic among four varied hip and knee arthroplasty clinics. | Retrospective study | Hip and knee arthroplasty outpatients | Telehealth visits via telehealth platform or telephone call | Telehealth utilization peaked during March and April of 2020 and has since reverted to near pre-pandemic levels. Younger patients and lower complexity visits such as postoperative or follow-up visits are more likely to use telehealth. |
| Burke et al, 2011 USA | To evaluate the utilization of a web-based multimedia patient-accessible electronic health record for patients with congenital cardiac disease. | Prospective study | Patients undergoing congenital cardiac surgery | Electronic health record after cardiac surgery | Users were found to access the system more often while in hospital than after discharge. Imaging data were viewed significantly more frequently than textual data. The adoption rate was high, and results suggest that the EHR could become a tool for health information exchange. |
| Calhoun, et al, 2017 | To explore possible sociodemographic disparities in access to the internet at home among veteran smokers. | Randomized trial | Veterans | Internet based smoking cessation program | Results indicate gaps in internet access and use among veterans and underscore the importance of improving accessibility of eHealth interventions for low-income, minority, and socially disadvantaged patients. |
| Chao et al, 2021 USA | To assess patterns of telehealth use across surgical specialties before and during the COVID-19 pandemic. | Statistical, descriptive, and sensitive analysis | Surgical outpatients | Video visit | Telehealth use grew across all surgical specialties in Michigan in response to the pandemic, but the use varied across surgical specialties. Telehealth use still remains substantially higher across all surgical specialties. |
| Chitungo et al, 2021 South Africa | To assess the use of digital health in sub-Saharan Africa | Rapid review | Patients in sub-Saharan Africa | Telephone, social media platform WhatsApp, virtual platforms, video calls | The most used mode was telephone. Benefits of using digital health services were medical service provision, connecting with patients, education, raising awareness of mental health issues, and toxicovigilance and infection control. Challenges contained lack of supporting telemedicine framework and policies, digital barriers, and patient and healthcare personnel biases. |
| Choi et al, 2021 USA | To investigate the concordance in diagnosis and management between initial telemedicine visits and subsequent in-person visits with laryngoscopy for laryngology-related complaints during COVID-19. | Retrospective cohort study | Patients of tertiary care center | Video visits for diagnosis | Concordance rates in diagnosis and management were high between the initial telemedicine visit and subsequent in-person visit with laryngoscopy for new patients. Telemedicine may still be a feasible alternative to provide suitable empiric therapy until laryngoscopy can be safely performed. |
| Coldebella et al, 2018 Australia | To assess the evidence relating to the use of telemedicine for providing health services to bariatric surgery patients. | Literature review | Bariatric surgery patients | Online education modules, video visits, mobile devices for store-and-forward or for teleconferencing | Review suggests that telemedicine may be a potential method for providing healthcare services to bariatric surgery patients. |
| Compton et al, 2021 USA | To study and increase the percentage of eligible adult CF patients who owned a home spirometer (HSs). | Controlled trial | Adult cystic fibrosis (CF) patients | Home spirometry monitoring and video visits | From March 2020 to September 2020 the number of patients seen via video visits performing spirometry at home increased substantially. The process of monitoring spirometry data with home devices via telemedicine was seen as reliable and sustainable. |
| Dayal et al, 2019 USA | To evaluate the association between access to telemedicine clinics and hospital utilization. | Retrospective cross-sectional study | Children with neurologic conditions | Video consultation | Pediatric neurology care through real-time, audiovisual telemedicine consultations was associated with lower hospital utilization compared to in-person consultations. |
| Dolezel et al, 2021 Czech Republic | To study the popularity of eHealth/mHealth services among young adults in the Czech Republic. | Questionnaire survey | Young adults | Health information seeking online, ePrescription services, doctor appointment organizers, recording/ monitoring vital signs | Health tutorial activities (i.e., acquiring information on diet, exercise, fitness) were the most common among the respondents, followed by health information seeking and medical services (i.e., ePrescription and doctor appointment organizers). Gender and existing health condition had impact on use. |
| Douglas et al, 2017 USA | To characterize telemedicine utilization among Medicaid enrollees by patients' demographic characteristics, geographic location, enrollment type, eligibility category, and clinical conditions. | Statistical analysis | Patients from 0 to 64 years of age | Telemedicine use, eg, video visits | Actual utilization of telemedicine in Medicaid programs was low. It was predominantly used to treat behavioral health diagnoses. |
| Elhadi et al, 2021 Libya | To assess the usability of telehealth services in Libya and to provide an overview of the current COVID-19 scenario. | Cross-sectional study | Libyan residents | Telehealth, eg, video visits | Study indicated high levels of usability and willingness to use the telemedicine system among the Libyan residents. However, internet connectivity and electricity issues could be a substantial barrier for many communities. |
| Gan et al, 2021 USA | To survey if new and follow-up patient visits for pediatric urology could be done effectively in a way that satisfied patient and parental expectations. | Questionnaire survey | Patients of pediatric urology | Telehealth, eg, video visits | Use of telemedicine proved a viable alternative method to provide care for pediatric urology patients. Families indicate a high degree of satisfaction with the technology in addition to time and cost savings. |
| Girgis et al, 2017 Australia | To test the feasibility and acceptability of PROMPT-Care (Patient Reported Outcome Measures for Personalized Treatment and Care). | Mixed methods approach | Cancer patients | PROMPT-Care system | Patients and oncology staff found the PROMPT-Care system to be highly acceptable, and the results suggest that it would be feasible to implement in an oncology setting. |
| Gonzalez et al, 2019 Australia | To summarize the study outcomes of the use of exploring the evidence for telemedicine in the clinical care of patients with heart failure and readmissions. | Review of randomized controlled trials | Heart failure patients | - Combination of Internet-based TM and telephone support.  - Automatic transfer of biometric data, generation of warning alarms, and alerts | Six papers supported and six others criticized the use of telemedicine for improving heart failure readmission. Patients had better rates of treatment with angiotensin-converting enzyme inhibitors/angiotensin II receptor blocker and beta-blockers, and telemonitoring and automatic transmission of vital signs were less utilized in comparison to studies in which telemedicine use was supported. |
| Halwas et al, 2017 Germany | To investigate Internet and eHealth usage with respect to eHealth literacy of cancer patients and their relatives. | Questionnaire survey | Cancer patients and their relatives | Internet usage | Internet usage was found to directly associate with younger age and better Internet connection. Younger participants were also more confident and capable of gaining medical knowledge through eHealth services. Regular use of eHealth services facilitated the decision-making process. Reading ability was associated with a better understanding regarding eHealth offers. |
| Hansen et al, 2018 Norway | To investigate the use of eHealth platforms (apps, search engines, video services, and social media sites) and associations with the use of provider-based health care visits among people with type 1 diabetes mellitus (T1DM). | Cross-sectional study | People with diabetes (T1DM) | Internet for searching information, as well as social media and video services | Positive association between use of search engines and specialist visits was found, and that people with T1DM are frequent users of eHealth, GPs, and specialist services. No evidence was found that eHealth reduces the use of provider-based health care. |
| Hansen et al, 2019 Norway | To investigate whether the use of eHealth might change patients' decisions regarding doctor-seeking behavior and whether information acquired from the internet was discussed with a doctor. | Cross-sectional study | People with diabetes | Use of internet information for decision making | Using information found via the internet had a significant impact on doctor-visiting decisions among people with diabetes, especially aged 18 to 39 years and with anxiety/depression. It is important that the information posted is of high quality and that the differences between internet-users are considered. |
| Harris et al, 2022 USA | To describe the transition of The Spot's buprenorphine service to telemedicine during the COVID-19 pandemic and one year treatment retention. | Descriptive and statistical analysis | People with opioid use disorder | Mobile clinic for buprenorphine treatment | Most patients (80.7%) remained engaged in treatment at one year, 16.0% were lost to follow-up, and 3.3% were deceased. Patients newly engaged via telemedicine were more likely to be female and white compared to those in-person services. |
| Javier-DesLoges et al, 2022 USA | To determine the odds of accessing telemedicine either by phone or by video during the COVID-19 pandemic. | Retrospective study | Patients with urologic condition | Telephone or video visits | Older Hispanic patients or patients with Medicaid insurance were less likely to access telemedicine during the pandemic. No differences were identified in telemedicine utilization according to patient age, sex, or training type. |
| Jiang et al, 2022 China | To explore the perceptions and experiences of older patients (and healthcare providers) in the application of telehealth and online health information for chronic disease management of chronic obstructive pulmonary disease. | Qualitative descriptive study with semi-structured interviews | Patients with chronic obstructive pulmonary disease | Usage of online health information | The ability of patients to understand health information should be considered while facilitating access to online health information. The role of health responsibility and user experience in older patients' participation and sustained use of telehealth and online health information needs to be emphasized. In addition, the complex social context is a determining factor to be considered. |
| Kao et al, 2016 USA | To study associations between availability of the Health Buddy Program (HBP) and all-cause mortality, hospitalization, hospital days, and emergency department visits. | Retrospective cohort study | Patients with heart failure | Heart Buddy Program is a device controlling eg,, vital signs and other health-related information | The use of HBP, a content-driven telehealth system coupled with care management, was associated with significantly better survival and reduced hospitalization in Medicare beneficiaries with heart failure. |
| Kesavadev et al, 2012 India | To assess the effectiveness, safety, and costs of the Diabetes Tele Management System (DTMS(®)). | Retrospective cohort study Research | Type 2 diabetes patients | Self-monitoring of blood glucose (SMBG) values and dose adjustments | DTMS, based on telemedicine follow-up and multidisciplinary care with SMBG-based monitoring, appeared to be safe and cost-effective in the intensive treatment of T2D without serious co-morbidities. |
| Knitza et al, 2020 Germany | To explore mHealth usage, preferences, barriers, and eHealth literacy reported by German patients with rheumatic diseases. | Questionnaire survey | German patients with rheumatic diseases | Mobile application for medication, internet for information search | Patients with rheumatic diseases seem to be eager to use mHealth technologies to understand their chronic disease despite low mHealth usage and competency. |
| Kong et al, 2021 USA | To assess patients' ability and willingness to utilize telemedicine as well as identify barriers to more widespread adoption of telemedicine in rheumatology. | Observational, cross-sectional study | Patients visiting a rheumatology clinic | Telephone, video visit | Age of patient was the most significant contributing factor in patient perception of telemedicine; older patients were less likely to consider that the needs of their rheumatology visit could be met over the phone or via videoconferencing. Patients with a greater commute to the clinic were more likely to utilize telemedicine consultations. |
| Kuipers et al, 2019 Netherlands | To assess the validity as well as patient-reported usability and acceptability of an electronic inhalation monitoring device (EIMD). | Observational study | Patients with respiratory diseases | Electronic inhalation monitoring device | Patients considered the EIMD to be acceptable and easy to use, but many hesitated to continue its use. Reminders and motivational messages were not appreciated by all users, and more user-tailored features were desired. |
| Kushalnagar et al, 2019 USA | To investigate deaf women's awareness of genetic testing and BRCA 1⁄2 genetic testing and evaluate deaf women's use of eHealth platforms for health‐related issues as well as relationship between awareness of genetic testing and use of eHealth platforms. | Questionnaire survey | Deaf women & hearing women | eHealth platforms (i.e., internet and social media sites) | Deaf women who had heard of genetic testing were more likely to use social networking sites to read or share information about medical topics and watch health-related videos. |
| Kuwabara et al, 2020 USA | To study how digital health technologies can be used for patient education. | Literature review | Different patient groups | Text messages, mobile apps, wearable devices, social media, telehealth (eg, telemonitoring), digital therapeutics, virtual and augmented reality, big data and machine learning | Use of digital health technology seemed to improve patient education and implementation of skills and behaviors that are essential for using digital health services. |
| Lancaster et al, 2018 Canada | To determine the impact of patients' use of eHealth tools on self-reporting adverse effects and symptoms that promote changes to medication use. | Systematic literature review | Patients using eHealth for medication changes | Web-based tool or portal, messaging tool, personal health or medical record, reminders, educational resources | Study suggested that the use of eHealth may improve patient symptoms and lead to medication changes. Patients generally found eHealth useful in improving communication with health care providers. Health-related outcomes among frequent eHealth users improved in comparison with individuals who did not use eHealth frequently. Implementation issues such as poor patient engagement and poor clinician workflow integration were identified. |
| Lanier et al, 2021 USA | To measure patient satisfaction with a cloud-based telemedicine platform. | Questionnaire survey | Patients of an adult clinic | Video visits | 46% of participants indicated a preference for future telemedicine visits, 77% would recommend use of telemedicine also to others. |
| Larsen et al, 2019 Denmark | To examine individual characteristics and health-care usage of patients who took up the targeted preventive programs in response to their personal digital health profile. | Cross-sectional study | Patients of general practitioners in two Danish municipalities | Personal digital health profile, electronic patient record data | A personal digital health profile seemed to motivate people with low self-efficacy to attend targeted preventive programs. Patients who had fair or poor self-rated health, a body mass index above 30, low self-efficacy, were female, non-smokers, or lead a sedentary lifestyle, were most likely to attend the programs. |
| Lattimore et al, 2021 USA | To examine demographic and socioeconomic differences in surgical patient telemedicine usage during the COVID-19 pandemic. | Cohort study | Surgical outpatients | Video visits, MyChart (an internet/ smartphone application- portal to access medical record and communication) | Compared to visits before the pandemic, telemedicine visits during COVID-19 were more likely to be with patients (especially women) from the least socioeconomically distressed communities with an activated MyChart and pay with non-government or commercial insurance. |
| Lazar et al, 2021 USA | To understand the impact that video telehealth has on outpatient visit volume and reimbursement for maintaining care. | Retrospective study | Patients at an academic department of surgery | Video visit | Use of video telehealth was rapidly adopted during COVID-19, which made up most patient visits during that time (61.3%). It was also found that video telehealth led to significant reimbursements and allowed patients in numerous states to receive care. |
| Liaw, et al, 2019 USA | To assess awareness, perceptions, and value of telehealth in primary care from the perspective of patients. | Cross-sectional study | Primary care patients | Video visit | Telehealth users considered live video to enhance access to healthcare and were less connected to primary care than nonusers of telehealth were. |
| Lin et al, 2021 USA | To examine trends in use of tele-buprenorphine and compare demographic and clinical characteristics in patients who received tele-buprenorphine versus in-person treatment only. | Retrospective cohort study | Veterans diagnosed with opioid use disorder (OUD) | Video visit | Use of telemedicine to deliver buprenorphine treatment in Veteran Health Administration increased 3.5-fold between 2012 and 2019 from 2.29% of buprenorphine patients in FY2012 (n = 187) to 7.96% (n = 1352) in FY2019 nationally. Tele-buprenorphine patients were less likely to be male or black, and more likely to live in rural areas. |
| Lin et al, 2019 USA | To identify and summarize studies examining the effectiveness of telemedicine interventions to deliver treatment for patients with substance use disorders | Systematic literature review | Patients with substance use disorders | Video visit | Most studies suggested telemedicine interventions were associated with high patient satisfaction and are an effective alternative, especially among patients required to travel relatively far for in-person treatment. Telemedicine interventions could be feasible across different substance use disorders. |
| Lonergan et al, 2020 USA | To analyze the change in video visit volume at the San Francisco (UCSF) Comprehensive Cancer Center in response to COVID-19 and compare patient demographics and appointment data. | Cross-sectional study | Cancer patients | Video visit | The proportion of video visits increased from 7%-18% to 54%-72%, between the pre- and post-COVID-19 periods (11 weeks) without any disparity based on race/ethnicity, primary language, or payor. |
| Looi et al, 2021 Australia | To investigate private psychiatrists' uptake of video- and telephone-telehealth consultations and total telehealth and face-to-face consultation in Q2 (April-June) of 2020 in comparison to Q2, 2019. | Longitudinal analysis | Private psychiatrist patients | Telephone and video visit | Combined telehealth and face-to-face psychiatry consultations rose during the Q2, 2020 by 14% compared to Q2, 2019 and telehealth was approximately half of this total. Face-to-face consultations in 2020 comprised only 56% of the comparative Q2, 2019 consultations. Most consultations were by telephone (short consultations) while video consultations comprised 38% of the total telehealth consultations. |
| Looi et al, 2021 Australia | To investigate private psychiatrists' uptake of video and telephone telehealth, as well as total (telehealth and face-to-face) consultations for Q3 (July-September), 2020 in comparison to Q3, 2019. | Longitudinal analysis | Private psychiatrist patients | Telephone and video visit | Combined telehealth and face-to-face psychiatry consultations rose during the Q3, 2020, by 14% compared to Q3, 2019, with telehealth 43% of this total. Most consultations were by telephone (short consultations) while video consultations comprised 42% of the total telehealth consultations. |
| McGrowder et al, 2021 Jamaica | To examine the use of telehealth services among breast cancer patients as well as satisfaction among patients and health care professionals. | Literature review | Breast cancer patients | Video consultation, therapy services, virtual cancer care delivery, telerehabilitation | Numerous benefits were found in using telehealth among breast cancer patients. Telehealth is eg, seen as cost effective, convenient and decreasing the travel burden on patients. Telehealth also comprises distant monitoring of the side effects of treatment, symptom management, emotional and psychological support as well as home-based individually tailored exercise programs. |
| Miller et al, 2021 USA | To explore the existing literature to identify research on pediatric home-based palliative care for children with serious illness delivered via telemedicine. | Scoping review | Children with serious illness in need of palliative care | Video consultation, video phone service | Telemedicine was found to be a promising strategy to increase access to palliative care for children with serious illness. However, the review found a need for more robust information describing implementation and effectiveness models, adaptation across care settings, and maintenance over time. |
| Moorhead et al, 2017 USA | To assess the efficacy and safety of "seeing" versus "not seeing" medication dose reminders regarding medication adherence and risk for overdose among patients with chronic diseases. | Post hoc analysis | Adults with uncontrolled hypertension and type 2 diabetes | Medication dose reminder | Digital device reminder messages were associated with a 16 ± 16% increase in medication adherence, especially in patients with lower adherence. No events of overdoses related to medication dose reminders occurred. |
| Motz et al, 2021 USA | To determine the feasibility of a directly supervised exercise training program delivered exclusively with telehealth. | Random clinical trial | Patients with nonalcoholic steatohepatitis | Audio-visual telehealth platform | No adverse events occurred. Body weight, body fat and waist circumference all improved with exercise. |
| Müskens et al, 2021 Netherlands | To evaluate the use of an eHealth platform and a self-management outpatient clinic in patients with rheumatoid arthritis. Effects on health-care utilization and disease activity were also studied. | Time series analysis | Patients with rheumatoid arthritis | eHealth platform | After implementation of the platform in April 2017, the percentage of patients using the platform was stable at ∼37%. On average, users were younger, more highly educated and had better health outcomes than average in the rheumatoid arthritis population. The number of outpatient clinic visits per patient decreased. |
| Newman-Casey et al, 2021 USA | To assess and compare the initial utilization, safety, and patient experience of tele-ophthalmology during the COVID-19 pandemic among in-person visits, video and telephone call visits as well as deferred visits | Cross-sectional study | Patients with eye disorders | Telephone or video visits | In-person participants were significantly older and worried about their eyesight more than those who had telephone, video call or deferred visits. More white participants had in-person visits than minority participants. Of all telephone or video call visits, 1.5% resulted in an in-person visit within 1 day, 2.9% within 2-7 days, and 2.4% within 8-14 days after the virtual visit. |
| Ng et al, 2022 USA | To examine factors associated with accessibility and utilization of telehealth during the COVID-19 pandemic. | Cross-sectional study | Older adults, ≥ 65 years | Telephone and video appointments | Disparities in accessibility of telehealth by sociodemographic status were observed. Those who had no access to internet and had not participated in video/voice calls/conferencing prior were less likely to report having access to telehealth. Among those offered telehealth services, 43.0% reported using telehealth services. Disparities were also observed between ethnic groups. |
| Norden et al, 2020 USA | To compare virtual and in-person visits to Stanford's ClickWell Care (CWC). | Longitudinal study | Patients' virtual and in-person visits to Stanford's Click Well Care -clinic | Virtual visits | More labs and images were ordered for in-person visits. Repeat visits were more likely after in-person visits. Visits for anxiety and depression were more frequent in virtual visits. |
| Nouri et al, 2020 USA | To determine which patient characteristics are associated with the use of patient-facing digital health tools in the United States. | Literature review | Patients using digital health tools | Any type of patient-facing digital health tool except patient portals | The review included 29 studies. Most studies examined smartphone apps and text messaging programs for chronic disease management. Overall, in most studies no association between patient characteristics and use was found. In some studies, white race and poor health status appeared to be associated with higher use. |
| O'Gorman et al, 2016 Canada | To assess how Ontario Telemedicine Network (OTN) utilization differed throughout the province. | Longitudinal study | Patients with mainly mental health issues | Video visits | There were 652,337 OTN patient visits in Ontario from 2008/2009 to 2013/2014. Most visits concerned mental health and addictions (61.8%). Utilization in other areas of care such as surgery, oncology, and internal medicine was highest in the rural north, whereas primary care use was highest in the urban south. |
| Perdue et al, 2021 USA | To examine the utility of telemedicine in a pediatric rheumatology clinic for 3 months during the COVID-19 pandemic. | Longitudinal analysis | Pediatric rheumatology patients | MyChart Epic Health Systems app on smartphone or tablet, which enabled video visits | Study indicated decrease in new patients utilizing telemedicine (60%) compared to the percentage of new patient office visits (84%) the previous year. There was no change in no-show rate between groups and patient characteristics were similar. |
| Poss-Doering et al, 2020 Germany | To evaluate user-reported experiences, perceptions, and perspectives, focusing on interpretation of web-based personal electronic health records (PEPA). | Semi structured guide-based interviews | Gastrointestinal cancer patients | Personal patient-controlled electronic health record; eHealth; nationwide implementation; continuity of care | Users indicated that usage of PEPA simplifies information sharing across care settings. Patients perceived benefits for involvement in treatment processes and continuity of care but worry about financing and functionalities of reduced versions. Physicians were hesitant and consider integration into primary systems critical for interoperability, anticipate technical challenges, as well as resistance from older patients and colleagues. |
| Possemato et al, 2013 USA | To assess whether participation in Behavioral Telehealth Center (BTC) services is associated with increases in healthcare utilization and decreases in symptoms based on behavioral health screening instruments. | Retrospective analysis | Veterans Using Behavioral Telehealth Center Services | Telephone | Participants utilized significantly more substance use and mental health treatment services and had significantly lower alcohol and depression screening scores post-BTC services compared with pre-BTC services. This study supports that BTC services are associated with increased healthcare utilization and decreased alcohol and depressive symptoms. |
| Powers and Buckner 2018 USA | To study access and quality of person-centered dementia care to patients and their caregivers in rural areas by providing distance dementia care and caregiver support with Clinical Video-Telehealth (CVT). | Retrospective analysis | Dementia patients and their caregivers | Video visits | Over a 3-year period 45 CVT encounters were performed on patient-caregiver dyads during which some 80% patients had dementia confirmed and 89% had serious medical comorbidities. Acceptance of the CVT encounter was 98%, with 8770 travel miles saved. |
| Pratap et al, 2020 USA | To evaluate the feasibility and utility of capturing real-world MS-related health data remotely using a smartphone app, "elevateMS," to investigate the associations between self-reported MS severity and sensor-based active functional test measurements, and the impact of local weather conditions. | Cohort study | Patients with MS | Smartphone app | The elevateMS study app captured the real-world experience of MS, characterized some MS symptoms, and assessed the impact of environmental factors on symptom severity. The study provides further evidence that supports smartphone app use to monitor MS with both active assessments and patient-reported measures of disease burden. |
| Quinton et al, 2021 USA | To determine whether broadband internet availability is associated with telemedicine adoption among a rural patient population. | Observational study | Adult patients in western Tennessee | Internet | Access to broadband internet is a determinant of access and utilization of telemedicine for patients in rural communities. |
| Radhakrishnan et al, 2013 USA | To explore the association of patient characteristics with re-hospitalizations of patients with heart failure during a 60-day period of telemonitoring following hospital discharge. | Longitudinal study | Patients with heart failure | Telemonitoring | In the study it was found that patients admitted with heart failure as a primary diagnosis had higher likelihood of cardiac-related re-hospitalizations than patients admitted with heart failure as a secondary diagnosis, despite using telehealth. |
| Radtke et al, 2021 USA | To determine if patient satisfaction is greater after delivering postoperative care via telemedicine following minimally invasive gynecologic surgery. | Randomized controlled trial | Female patients after gynecologic surgery | Audio or video visits using smartphone | Postoperative care via telemedicine after gynecologic surgery seemed to result in higher patient satisfaction. |
| Rahman et al, 2021 Bangladesh | To examine gender disparity among telehealth usage during the COVID-19 pandemic in 2020. | Cross-sectional study | Bangladeshi outpatients from urban and rural areas | Telehealth visits | The analysis showed that male patients had a higher dependency on telehealth. It was also found that the youngest age group, 16-25, had the highest dependence on telehealth, and the lowest dependence was among the oldest age group of 45 years and above. |
| Schiaffini et al, 2016 Italy | To compare the long-term effect on glucometabolic control of eHealth intervention and traditional care in Type 1 diabetes (T1DM) SAP-treated adolescents. | Longitudinal study | Patients with Type 1 diabetes | Web-based platforms for telemonitoring and transmission of data | The study demonstrated that telemedicine added to SAP therapy significantly improves Type 1 diabetes adolescents’ compliance in disease self-management. |
| Sengpiel et al, 2010 Germany | To explore use of home spirometry with Bluetooth data transfer in outpatient lung transplant recipients. | Randomized controlled trial transplantation | Outpatients after lung transplantation | Home spirometry with Bluetooth for data transfer | Adherence to home spirometry was 97.2% in the Bluetooth group and 95.3% in home spirometry alone. Median time to first consultation and frequency of consultation did not differ significantly. Patients in the Bluetooth group reported less anxiety. |
| Speier et al, 2018 USA | To evaluate the adherence rates using a consumer-grade continuous-time heart rate and activity tracker, and to utilize the information provided by the device to identify information about a patient's state. | Longitudinal study | Mid-risk cardiovascular patients | Activity tracker in mobile phone for monitoring | Using continuous-time activity trackers with heart rate monitors can be effective in a telemonitoring application, as patients had a high level of adherence (90.0% median) and low attrition (0.09% decrease per day) over a 90-day period. Data correlated significantly with clinical patient surveys identifying patients in need of intervention. |
| Steventon et al, 2016 Great Britain | To assess the effects of a home-based telehealth intervention on the use of secondary healthcare and mortality. | Observational study | Patients with chronic obstructive pulmonary disease, heart failure or diabetes | Remote monitoring for exchange of medical data between patients and healthcare professionals | Telehealth patients were more likely to experience emergency admission or death, and more likely to have outpatient attendances. Mortality rates were similar between groups. It seemed that telehealth was not associated with a reduction in secondary care utilization. |
| Sultan et al, 2020 USA | To evaluate the feasibility and patient satisfaction associated with virtual visit utilization in pediatric spinal deformity (PSD) patients in comparison to general pediatric orthopedic indications. | Observational study | Patients with pediatric spinal deformity | Virtual visits to evaluating patients, and sharing data (images, laboratory values) | Virtual visit patients were older than average PSD patients (15±3.7 yrs) and had longer visits than their general pediatric orthopedic counterparts. Virtual visit patients demonstrated high satisfaction with surgeon performance and overall satisfaction. 80% of all virtual visits were conducted over mobile devices. Wait time was less for virtual visits relative to subsequent office visits. |
| Suman et al, 2019 Netherlands | To assess the effectiveness and cost-utility of a multifaceted eHealth strategy compared to usual care in improving patients' back pain beliefs, and in decreasing disability and absenteeism. | Cluster randomized trial | Patients diagnosed with non-specific low back pain | Mobile, website, digital newsletters, and social media platforms sharing information, video messages | The study showed no differences between groups in back pain beliefs, disability, or absenteeism, but showed promising cost-utility results based on quality adjusted life years. |
| Thakar et al, 2018 India | To evaluate the cost-effectiveness of telemedicine consultations for follow-up care of neurosurgical patients. | Cost-effectiveness analysis | Post-neurosurgical care patients | Skype teleconference for consultation | Telemedicine was found to dominate the in-person care strategy by providing more effective and less expensive follow-up care for remote post-neurosurgical care. |
| Townsend et al, 2015 Canada | To examine the challenges in medical encounters as roles and relationships shift and apply a conceptual framework of relational ethics to examine explicit and nuanced ethical dimensions emerging in patient-health care professional communication. | Semi structured discussion | Patients with chronic conditions (and healthcare professionals) | Internet for information searching | Patients reported making Internet searches via general health-related websites (eg, universities, health organizations, non-profit disease-oriented organizations) aimed at both patients and health care professionals. Patients also reported using personal websites and blogs, chat rooms, and online links to medical test results. Health care professionals reported finding information to a lesser extent, and resources were largely limited to health- and professional-oriented websites. |
| Tran et al, 2020 USA | To explore the feasibility and acceptability of collecting electronic patient-reported outcomes (ePROs) using validated health-related quality of life (HRQoL) questionnaires. | Mixed methods study | Prostate cancer patients | Digital health app collecting electronic PROs (ePROs) | Nearly all patients reported that using the smartphone app is easier than or equivalent to paper-and-pen. Most participants self-identified as having a high digital literacy level, and only a few participants identified as having a low digital literacy level. Interviews emphasized the value of emotional support and wellness in cancer treatment, rise in social patient advocacy in online patient communities and networks, concerns over privacy, and desire for personalized engagement tools. |
| Vakkalanka et al, 2021 USA | To evaluate the association between telehealth encounters and time to discontinuation of buprenorphine treatment when compared to traditional in-person visits and to evaluate eg, in-person and telehealth treatment. | Retrospective cohort study | Veterans diagnosed with substance use disorder (SUD) | Video encounters | Compared to in-person encounters, treatment discontinuation was lower for telehealth in substance use disorder and mental health. Risk of treatment discontinuation appeared to be lower among those with telehealth only compared to in-person only for both SUD and for mental health. |
| van Zelst et al, 2021 Netherlands | To explore the impact of healthcare professional involvement on the adherence of patients to an eHealth platform. | Observational cohort study | Patients with Chronic Obstructive Pulmonary Disease | eHealth platform | COPD patients used the eHealth platform more frequently in a blended care setting (with health care professional) compared to patients who used the eHealth platform independently. |
| Wade and Cartwright, 2012 Australia | To investigate acceptance of in-home telehealth by frail older adults and carers of the Transition Care Program (TCP) and evaluates telehealth acceptance as a predictor for usage compliance. | Quasi-randomized controlled clinical trial | Older adults and carers of the Transition Care Program | Telehealth monitoring for vital signs | Before being trained in and using telehealth, most participants and carers demonstrated acceptance of the technology. This acceptance was also reported post-TCP (up to 12 weeks of usage). The perceived ease of use of the telehealth equipment increased significantly from pre-telehealth training and usage to post-TCP. |
| Waibel et al, 2017 USA | To describe specialty-specific usage or patient satisfaction regarding synchronous or “real-time” telehealth at the regional military hospital level. | Retrospective review | Patients at military hospital | Telehealth visits for consultation | 2,354 synchronous telehealth encounters were conducted for 1,886 unique patients. Clinic utilization varied between specialties as well as whether a specialty-trained patient presenter was preferred. |
| Wati et al, 2021 Thailand | To determine the telemedicine usage and factors contributing to glycemic control in type 2 diabetes mellitus (T2DM) patients during the COVID-19 pandemic. | Cross-sectional study | Type 2 diabetes mellitus (T2DM) patients | Mobile health applications (m-Health) or other platforms (eg,, WhatsApp) usage for consultation | Of 264 patients, only 19.2% used telemedicine and 60.2% had poor glycemic control during the pandemic. Thus, based on this study, glycemic control was not optimal during the COVID-19 pandemic. |
| Wattanapisit et al, 2020 Thailand | To identify usability and utility of eHealth for tailored physical activity counselling introduced in primary health care settings. | Scoping review | Primary health care patients | eHealth usage for counselling | The eHealth tools covered a wide range of counselling domains from stand-alone physical activity to multiple health behaviors. The study presented mixed findings on the usability and utility of eHealth for physical activity counselling. Technical problems and complexity of the programs were seen as barriers to usability. |
| Wegermann et al, 2021 USA | To determine if disparities in race or socioeconomic status exist among patients utilizing telehealth visits during COVID-19. | Retrospective cohort study | Adult patients of hepatology clinics a | Video/phone visits for counselling | According to the study, disparities in use and suboptimal use (phone versus video) remain for vulnerable populations including those that are older, non-Hispanic black, or have Medicare/Medicaid health insurance. |
| Whealin et al, 2016 USA | To identify the types of eHealth tools that veterans with Post-Traumatic Stress Disorder (PTSD) and comorbid chronic medical conditions (CMC) use, understand how they currently use eHealth technology to self-manage their health care needs, and identify new eHealth resources for better health management. | Sequential, mixed methods study | Veterans With Post-Traumatic Stress Disorder and Comorbid Health Conditions | Electronic personal health record system to self-manage their health care needs | The study suggested that veterans who use the Web are eager to incorporate eHealth technology into their care and self-management activities. |
| Wong et al, 2021 Australia | To assess glycemic control of patients with diabetes attending telehealth consultations in 2020 compared to face-to-face reviews prior to the COVID-19 pandemic. | Retrospective study | Patients with diabetes | Phone or video visit for consultation | The attendance rate from April to September 2020 for telehealth consultation at the diabetes services was higher than in 2019. The study showed that for patients who received care via telehealth consultations during the COVID-19 lockdown, their glycemic control was slightly better, and unplanned admission rates were not higher compared to the pre-COVID-19 period. |
| Woo et al, 2016 USA | To evaluate a spinal cord injury (SCI) disease management protocol clinical content and to identify issues for broader implementation across the Veterans Affairs SCI System of Care. | Semi-structured qualitative survey | Patients with spinal cord injuries and disorders | Data messaging device to assess symptoms, educational needs, and self-management behavior recommendations | Patients reported that the program was most beneficial for newly injured patients recently discharged from acute rehabilitation that live far from specialty spinal cord injury care facilities. |
| Yadav et al, 2019 Australia | To evaluate the effectiveness of digital health supported targeted patient communication versus usual provision of health information. | Systematic review and meta-analysis | Older people, aged > 50 with a fragility fracture | From voice call to applications of multimedia technologies | Digital health supported targeted patient communication with primary care physician involvement could be twice as effective as usual care in prevention of secondary fractures among patients with fragility fractures. |
| Yi et al, 2015 USA | To assess self-blood monitoring | Randomized clinical trial | Medically underserved and black and Hispanic participants | Blood pressure remote monitoring | Self-blood pressure monitoring was not shown to improve control over usual care in largely minority, urban population. Results indicate that underserved and minority groups may have additional barriers to achieving blood pressure control beyond access to the monitor. |
| Zhang et al, 2021 China | To explore the utility of deep brain stimulation (DBS) telemedicine in the management of patients with movement disorders from January 2019 to March 2020 during the COVID-19 outbreak. | Retrospective study | Outpatients with Parkinson’s disease or dystonia in China | Video connection service | The results showed that the number of telemedicine sessions requested, and the number of patients examined increased. Most (89%) tele-programming adjustment sessions were experienced by the patients as satisfactory. |
